# Containing the spread of COVID-19 in Ethiopia

**DOI:** 10.7189/jogh.10.010369

**Published:** 2020-06

**Authors:** Zemzem Shigute, Anagaw Derseh Mebratie, Getnet Alemu, Arjun Bedi

**Affiliations:** 1 International Institute of Social Studies, Erasmus University Rotterdam, the Netherlands and Addis Ababa University, Addis Ababa, Ethiopia; 2 School of Public Health, Addis Ababa University, Addis Ababa, Ethiopia; 3 Institute of Development and Policy Research, Addis Ababa University, Addis Ababa, Ethiopia; 4 International Institute of Social Studies, Erasmus University Rotterdam, the Netherlands

Ethiopia has a low although rising number of confirmed COVID-19 cases. Despite these low figures, stringent measures have been implemented since mid-March. In this viewpoint we describe the prevention and preparation measures taken in Ethiopia and comment on the consequences, challenges and strengths of the measures, keeping in mind the Ethiopian context.

## LOW FIGURES – A WINDOW OF OPPORTUNITY

Of the 48 countries in Sub-Saharan Africa (SSA), all have reported cases of COVID-19 infection. At the time of writing, the SSA region has close to 76 500 confirmed COVID-19 cases with 1748 deaths. These figures account for a small proportion of global COVID-19 infections (1.4%) and an even smaller proportion of deaths (0.51%) [1].

This may in part be attributed to limited testing and poor reporting systems, resulting in a distorted or perhaps more dangerously an overly optimistic picture. Alternatively, it may reflect the relatively lower integration of such countries in the world economy and the earlier imposition of lockdown-style measures. While it may only be a matter of time before the epicenter of the pandemic shifts again, at least at the moment, countries with the most vulnerable health care systems in the world have a window of opportunity to prepare and to potentially prevent community spread of the virus.

In Ethiopia, the vital fear of dealing with the virus in the context of weak health systems and a vulnerable economy, energized the country’s leadership and led to the early imposition of stringent measures.

In Ethiopia, measures were adopted on March 16 and further sharpened on March 20 when there were only 5 confirmed cases. On April 10, a five-month state of emergency was declared. In terms of the stringency of the measures, at least on paper, our calculations show that the preventive measures adopted by Ethiopia place it in the most stringent category. Ethiopia has a score of 85 while India is at the maximum of a 100 [2].

At the time of writing (May 25, 2020), there are 655 confirmed cases based on 83 854 tests. There have been 159 recoveries and five deaths. Almost all the confirmed cases are restricted to urban areas (21% of the population) with a majority of cases (67%) occurring in the capital, Addis Ababa [3].

## PREVENTION AND PREPARATION

### Prevention

Taking cues from the international response the government organized itself efficiently, in a so-called, whole-of-government approach to economic and emergency management and quickly adopted a raft of preventive measures.

These include:

- **International travel** – isolation of passengers arriving from international destinations and suspension of flights,
- **Quarantine** – more than 16 000 people have been placed in quarantine for 14 days with 27 universities serving as quarantine center,
- **Spread of World Health Organization recommended practices** – such as frequent hand washing, avoiding handshakes, elbow sneezing and coughing through mass media,
- **Free provisions** – toll free telephone lines for information and free provision of sanitary items such as soap and hand-washing gels to targeted groups in Addis Ababa,
- **Closures** – of schools, universities, bars and nightclubs; suspending public gatherings and meetings and issuing stay-at-home orders for all but necessary staff,
- **Subsidized** – internet and voice package offered by Ethio telecom,
- **Mass disinfection** – of critical urban locations,
- **Avoiding overcrowding** – by reducing the maximum number of passengers in trains, taxis and buses to half their capacity,
- **Complete transport lockdown** – in some regions of the country except for carriage of essential supplies,
- **Release of prisoners** – release of around 4,000 pr,isoners who committed minor offences and/or were to be soon released
- **Postponed** – perhaps most notably, national elections scheduled for August 2020 have been postponed.

While these measures are similar to those taken in other parts of the world, a key difference is that a majority of Ethiopians (79%) live in rural areas with weak transportation and communication links.

To reach these areas, risk communication and community engagement task forces have been established at the lowest administrative units and at health facilities. These units involve the country’s 42 000 health extension workers, two per village, who undertake the task of household and individual level sensitization and awareness creation. The social distancing measures in rural areas relate to agricultural marketing, avoidance of social gatherings while at the same time continuing daily agricultural tasks such as *belg* (autumn) crop season plantation. The country’s key social protection program, the productive safety net program (PSNP), which requires community labor contributions, has been re-oriented to individual based activities to avoid social contact.

### Preparation

At the onset of the crisis, virus testing facilities in the country were limited. With international support these have been rapidly ramped up. Currently there are about 24 testing laboratories in the country, capable of performing more than 5600 tests a day. About 18 000 health professionals, including students and retirees have been mobilized of which 5000 began serving immediately and a large exhibition hall in Addis Ababa has been refurbished as a treatment center.

To prepare the country’s health system, international help has been actively solicited. The government’s resource mobilization and health emergency teams are distributing testing kits and personal protective equipment donated by Chinese billionaire Jack Ma. The World Bank has provided US$ 82 million to support the country’s health care needs and the International Monetary Fund approved US$ 411 million. Financial and material resources are also being obtained through Ethiopian nationals and through the 2 million strong Ethiopian diaspora [4]. The foreign ministry has issued a request to all Ethiopian missions to raise funds and buy critical medical equipment and ship to the country.

The government’s health care team has been working with Chinese health care experts to enhance the capacity and expertise of its health care system. On April 16, a team of Chinese Anti-pandemic medical experts arrived in Addis Ababa.

**Figure:**
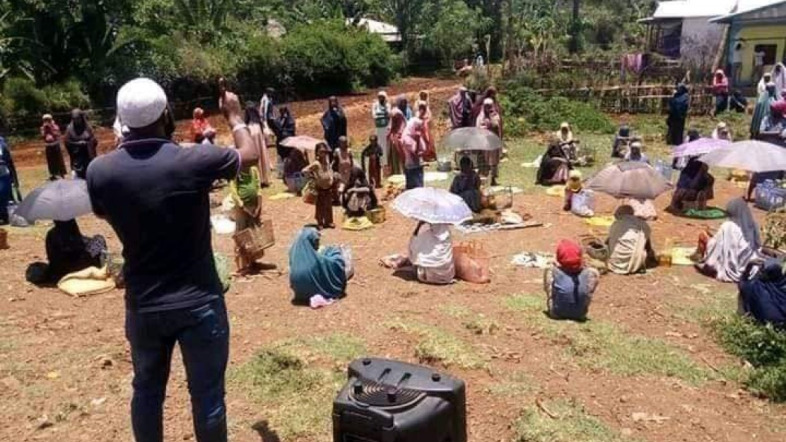
Photo: Awareness creation on “corona-cognizant” marketing of goods by COVID-19 village task force member, SNNPR region, 11 April 2020 (used with the permission of Ahmed Mohammed Ali).

### Consequences, challenges and strengths

**Balance:** The various preventive measures are transforming the health shock into a wider socio-economic shock, especially for the service sector and those sectors that are internationally oriented. There has been a decline in international remittances, tourism has dried-up, the country’s airline is experiencing sharp losses. Demand for horticultural exports - especially flowers, which tend to employ a substantial proportion of female workers has evaporated.

However, a balance has been maintained. The lockdown measures while seemingly stringent are not being strictly enforced. Economic activities are continuing albeit at a lower level and in a country with a large informal sector and reliance on day to day income, a deliberate decision has been taken not to be heavy-handed with a view to restricting a sharp increase in vulnerability.

Measures to mitigate the economic effects of the crisis have been put in place. Rents on government-owned property have been reduced and business owners and individuals have also been asked to take similar measures. To ensure food security – more than 1200 food banks have been set up for the urban poor in Addis Ababa. The government is pushing households who can afford it to provide one meal per day for a poor household so as to reduce the possibility of civil unrest.

The country’s main social safety net (8 million beneficiaries), the PSNP which caters to rural areas is working actively to shield the vulnerable. In rural areas, guided by development agents, economic activities, especially farming and marketing of produce is continuing in a “corona-cognizant” manner. At the moment the agricultural supply chain has remained stable and there are no reports of food shortages in urban areas.

**Tactile nation:** Preventing social contact in a tactile country such as Ethiopia is very difficult. Ethiopian social and religious practices and daily culture entail physical contact, embodied for example in communal eating habits and in the way of greeting. The importance of community, both culturally and in the country’s development strategy make it hard to respect social distancing. Even if there are efforts to implement ‘social distancing’ and to encourage ‘stay at home’ principles, these are most apparent only in Addis Ababa. In most other major towns of the country, markets remain crowded and life continues almost as usual although attempts are being made to enforce the state of emergency and rein in weddings and other festivities.

**Challenged health system:** Despite the various measures taken to prepare the health system, it is unlikely that it will be able to handle patient surges, further underlining the need for preventive measures. While access to health care has sharply increased in the last ten years and a substantial number of households are covered by a community-based health insurance scheme introduced in 2011, resources are limited. Ethiopia has a total of 557 mechanical ventilators and 570 intensive care unit (ICU) beds for a population of 110 million.

Driven by the fear of contracting the virus through health facilities, there has been a sharp decline in the use of outpatient and inpatient services at public hospitals. For instance, in Addis Ababa University’s Black Lion hospital, patient numbers have declined and there has been an increase in absenteeism amongst health professionals.

**Young:** Ethiopia is a young country with 40% of its population aged 0-14 and only about 8% aged 55 and above. Given the epidemiological profile of the confirmed cases and deaths in the Global North, this may seem positive. However, the country’s young population is not very well nourished, with stunting in 38% of children aged 0-5 and undernourishment of 22% of women aged 15-49 [5].

**Population density and distance:** The dangers of community transmission loom larger in urban areas with high population density, and especially in Addis Ababa (6516 inhabitants per km^2^). However, the bulk of the country has a substantially lower population density: the most populous region Oromia has a population density of 124 people per km^2^. The low population density in rural areas and relatively poor transportation infrastructure which restricts internal mobility might limit the spread of the virus in the rural hinterland.

**Experience:** Ethiopia is no stranger to widespread shocks, although hitherto, most of these have been weather related (droughts and famine) and often confined to rural areas. Since the disastrous 1983-1984 drought which claimed more than a million lives, the government has strengthened its ability to withstand shocks rather than rely on humanitarian appeals.

In the most recent drought in 2016, the government supported 18.2 million people, or 20% of the total population, through food or cash transfers with the PSNP playing a leading role. The institutional infrastructure involving strong community outreach provides a platform to reach the most vulnerable rural populations and can later serve as a conduit for providing economic support and recovery.

## CONCLUSION

The government has moved swiftly and prudently and rolled out a range of measures. On paper, the measures are stringent. However, deliberately, keeping in mind the country’s fragile economy, and the social and economic conditions of its citizens, the lockdown has not been heavy-handed. A good balance has been maintained, and economic activities, especially agriculture and industry, have continued with a view to maintaining food security and preventing unrest.

The country’s early response, its young population, low population density in rural areas, experience in handling large scale crises, dense network of community workers are positive aspects in the fight against the virus. However, these are pitted against a weak health system, poor nutritional status, lack of access to proper hygiene and sanitation and densely populated urban areas.

While preparatory measures need to continue, the country’s best hopes lie in its strategy of early imposition and continued adherence, if not strengthening of preventive measures, to avoid widespread community transmission of the virus.
